# HILIC-Enabled ^13^C Metabolomics Strategies: Comparing Quantitative Precision and Spectral Accuracy of QTOF High- and QQQ Low-Resolution Mass Spectrometry

**DOI:** 10.3390/metabo9040063

**Published:** 2019-04-02

**Authors:** André Feith, Attila Teleki, Michaela Graf, Lorenzo Favilli, Ralf Takors

**Affiliations:** Institute of Biochemical Engineering, University of Stuttgart, Allmandring 31, 70569 Stuttgart, Germany; andre.feith@ibvt.uni-stuttgart.de (A.F.); teleki@ibvt.uni-stuttgart.de (A.T.); michaela.graf@ibvt.uni-stuttgart.de (M.G.); lorenzofavilli@outlook.com (L.F.)

**Keywords:** LC-QTOF-HRMS, LC-QQQ-MS/MS, HILIC, Quantitative metabolomics, IDMS, Isotopic distribution, Spectral accuracy, ^13^C-tracer studies, *Corynebacterium glutamicum*

## Abstract

Dynamic ^13^C-tracer-based flux analyses of in vivo reaction networks still require a continuous development of advanced quantification methods applying state-of-the-art mass spectrometry platforms. Utilizing alkaline HILIC chromatography, we adapt strategies for a systematic quantification study in non- and ^13^C-labeled multicomponent endogenous *Corynebacterium glutamicum* extracts by LC-QTOF high resolution (HRMS) and LC-QQQ tandem mass spectrometry (MS/MS). Without prior derivatization, a representative cross-section of 17 central carbon and anabolic key intermediates were analyzed with high selectivity and sensitivity under optimized ESI-MS settings. In column detection limits for the absolute quantification range were between 6.8–304.7 (QQQ) and 28.7–881.5 fmol (QTOF) with comparable linearities (3–5 orders of magnitude) and enhanced precision using QQQ-MRM detection. Tailor-made preparations of uniformly (U)^13^C-labeled cultivation extracts for isotope dilution mass spectrometry enabled the accurate quantification in complex sample matrices and extended linearities without effect on method parameters. Furthermore, evaluation of metabolite-specific *m+1*-to-*m+0* ratios (ISR_1:0_) in non-labeled extracts exhibited sufficient methodical spectral accuracies with mean deviations of 3.89 ± 3.54% (QTOF) and 4.01 ± 3.01% (QQQ). Based on the excellent HILIC performance, conformity analysis of time-resolved isotopic enrichments in ^13^C-tracer experiments revealed sufficient spectral accuracy for QQQ-SIM detection. However, only QTOF-HRMS ensures determination of the full isotopologue space in complex matrices without mass interferences.

## 1. Introduction

Systems metabolic engineering relies on proper, data-driven metabolic models asking for reliable identification and quantification of endogenous metabolic pools under in vivo conditions. Often, ^13^C metabolic flux analysis is applied which inherently needs detailed information about isotopic labeling distributions of intracellular metabolites either in steady states [1,2,3,4] or in transient time series [1,3,5]. The first is usually achieved by targeted ^13^C-tracer studies (^13^C-based Metabolic Flux Analyses, MFA) whereas the second demand non-stationary labeling approaches (^13^C-NMFA) measuring time-resolved isotopic enrichments in conjunction with absolute concentrations to estimate reaction rates. Considering the complexity of metabolic networks, comprising an enormous number of intermediates covering a wide chemical diversity and concentration ranges up to 10 orders of magnitude (pM-mM), quantitative and exhaustive approaches with high precision requirements are still a great analytical challenge [6,7,8].

Current analytical methods range from nuclear magnetic resonance spectroscopy (NMR) to electrospray ionization mass spectrometry (ESI-MS) mainly coupled to gas (GC) or liquid chromatography (LC) [9,10,11,12,13,14]. For comprehensive metabolic studies, LC-ESI-MS platforms offer significantly higher sensitivity compared to NMR [15]. Besides, usually no sophisticated derivatization procedures are required, as needed for GC, leading to additional isotopic backgrounds, multiple chromatographic peaks, and low signal responsivities of charged or polar compounds [1,16]. In this regard, hydrophilic interaction liquid chromatography (HILIC) is increasingly favored, as it enables a direct and simultaneous separation of polar intermediates of central carbon metabolism, energy regeneration, and anabolism via a single analytical platform and it exhibits wide compatibility with ESI-MS detection [13,17,18,19]. ^13^C-tracer-based studies imply further analytical demands on the need to measure metabolic isotopomers containing *n* carbon atoms with 2*^n^* possible labeling states [20]. However, using mass spectrometry, the resolution of distinct labeling positions in metabolites is intrinsically limited. Instead, labeled fractions differing in isotopic composition independent of position (isotopologues) are typically identified. Whereas this information is basically sufficient to perform ^13^C MFA of networks with known reaction stoichiometry [2,5,12], the accurate detection of isotope mass spectra in complex sample matrices still demands high spectral accuracy and high resolution of applied LC-MS platforms [21,22]. Even though triple quadrupole tandem mass spectrometry (QQQ-MS/MS) provides excellent sensitivity and specificity, it also has limited unit mass resolution and does not provide structural information and identification of non-targeted compounds [23,24]. By contrast, quadrupole time-of-flight high-resolution mass spectrometers (QTOF-HRMS) exhibit excellent mass accuracies (up to ±5 ppm) and also provide elemental composition and structural elucidation of non-targeted compounds [24,25]. Complementary approaches using HRMS for interference-free measurements of isotopic mass spectra and MS/MS for precise quantification of corresponding absolute concentrations [26,27] are typically time-consuming and require multiple analytical MS platforms, which may not always exist in interdisciplinary research groups. 

Here, we employ an optimized alkaline HILIC method [13] as the basis for a comparative LC-QQQ and LC-QTOF study quantifying metabolite pool concentrations and isotopologue distributions in ^13^C-labeled *Corynebacterium glutamicum* extracts. Seventeen key intermediates represent a cross-selection of the microbial central carbon metabolism, comprising the glycolysis (G6P, F6P, 2/3PG, PEP), the citric acid cycle (Suc, aKG, Mal) and the pentose phosphate pathway (Pen5P, S7P), as well as amino acids (Phe, Pro, Val, Ala, Gln, Ser, Glu, Asp). Particular focus will be given to the systematic evaluation of metabolite-specific calibration ranges, repeatability, signal sensitivities, determination limits, and measuring precision using QQQ-MRM and QTOF-MS detection with pre-optimized settings. The applicability and reliability of isotope dilution mass spectrometry (IDMS) for absolute metabolite quantification protocols will be investigated using tailor-made uniformly (U)^13^C-labeled *C. glutamicum* cultivation extracts. Furthermore, the spectral accuracy of both MS systems will be evaluated with respect to the natural isotopic distributions of relevant compounds using non-labeled standard mixtures and multicomponent intracellular *C. glutamicum* extracts. Precision and accuracy of QQQ and QTOF mass detection will be finally assessed by comparative measurements of non-stationary and stationary labeled endogenous extracts from a ^13^C-tracer experiment in continuous *C. glutamicum* cultivation.

## 2. Results

### 2.1. Optimization of Chromatographic Conditions and ESI-MS Parameters

Optimized chromatographic conditions and electrospray ion source (ESI) parameters were based on previous HILIC-MS/MS studies for targeted quantitative profiling of more than 50 key metabolites in endogenous cellular extracts [13]. QQQ-MS/MS studies of metabolite-specific signal responsivities optionally using isotope dilution mass spectrometry (IDMS) were optimized by preliminary flow injection analysis (FIA, column bypassing) of standard solutions in multiple reaction monitoring (MRM) mode. Precursor-to-product ion transitions, associated MS/MS settings, and source parameters were selected regarding expected elution conditions and maximal ESI-MS responsivities of the targeted non- and fully labeled metabolites (Table S1). Analogously, QTOF-HRMS experiments were performed in MS mode with separately pre-optimized metabolite-specific MS parameters and source conditions (Table S2). The QTOF system was tuned in extended dynamic range (EDR) mode (2 GHz) to achieve the broadest linearity range possible.

For targeted ^13^C-labeling analysis, the approach was extended by transfer of the optimized MRM parameters to selected ion monitoring (SIM) mode for the QQQ-system. Precursor masses and associated MS/MS settings were adapted concerning potential labeling states of corresponding ^13^C_n_ isotopologues (*m+n*). Additional targeting for hypothetic contiguous labeling states enabled the detection of mass spectral overlaps by interfering metabolites (Table S3A,B). Acquisition of high-resolution ^13^C-isotopologue data was performed in positive and negative ESI mode with global MS settings utilizing the QTOF system, tuned in high-resolution (HR) mode (4 GHz). Metabolite-specific mass extraction parameters were used for data analysis (Table S4).

### 2.2. Metabolite-Specific Linearity Ranges and Sensitivities 

Linear dynamic ranges of non-labeled metabolites were comparatively analyzed over almost six orders of magnitude (5 nM to 800 µM, 5 µL sample injection) by a QQQ-MS/MS (MRM mode) and a QTOF-HRMS (MS mode) platform using optimized conditions and parameters. Concentration ranges were considered linear when the squared correlation coefficient (*R*^2^) was better than 0.99 for the average of four measurements (*n* = 4). 

**Figure 1:**
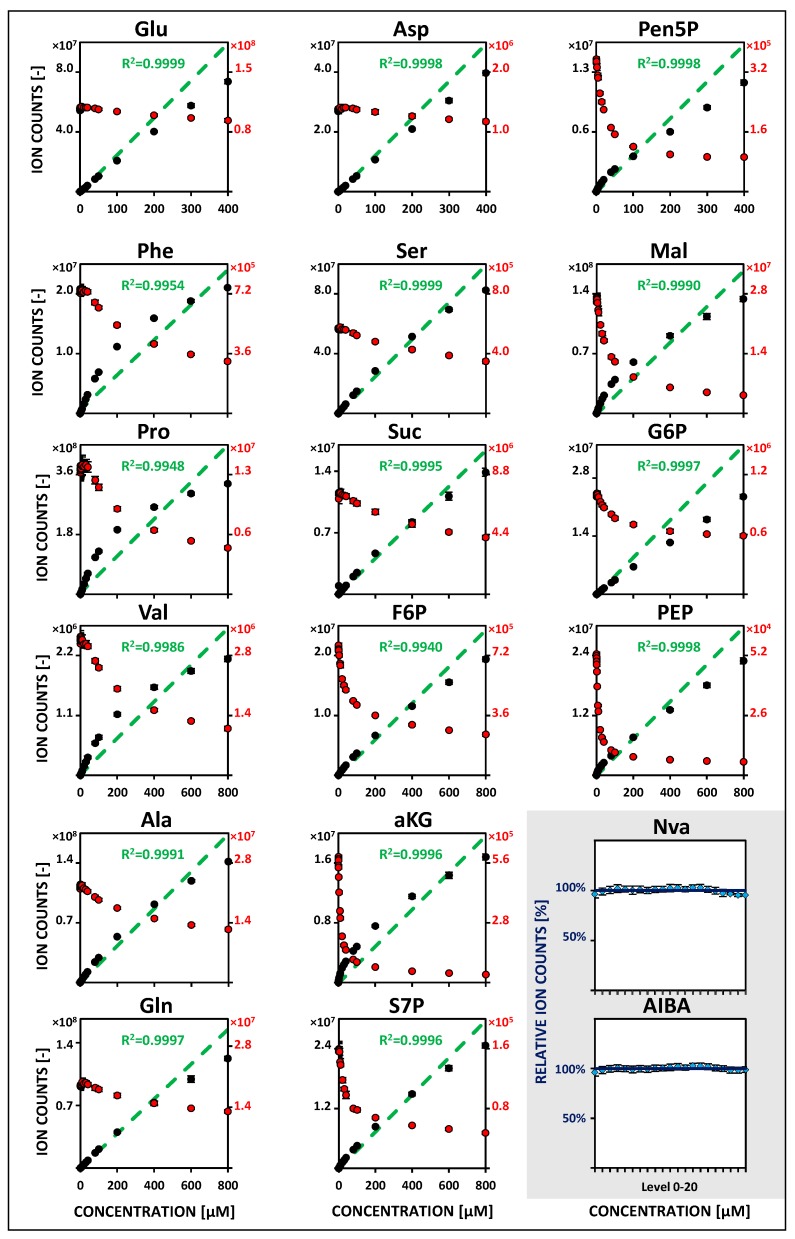
HILIC-based QQQ-MS/MS studies in MRM mode of metabolite-specific signal responsivities using U^13^C isotope dilution (IDMS). Metabolite-specific calibration curves in the extended concentration range (5/10 nM to 400/800 µM, 5 µL sample injection). Mean values of non-labeled reference standards (black) and analogous U^13^C-isotopologues (red) are based on four freshly prepared (24 h) replicates (*n* = 4). Linear regression of IDMS normalization (green) is shown with associated correlation coefficients (*R*^2^ > 0.99). Non-labeled internal standards (blue) are additionally considered for monitoring of instrumental fluctuations (*n* = 4). See Table S5 for related regression parameters. See Tables S1–S4 for the full names of abbreviated metabolites.

**Figure 2:**
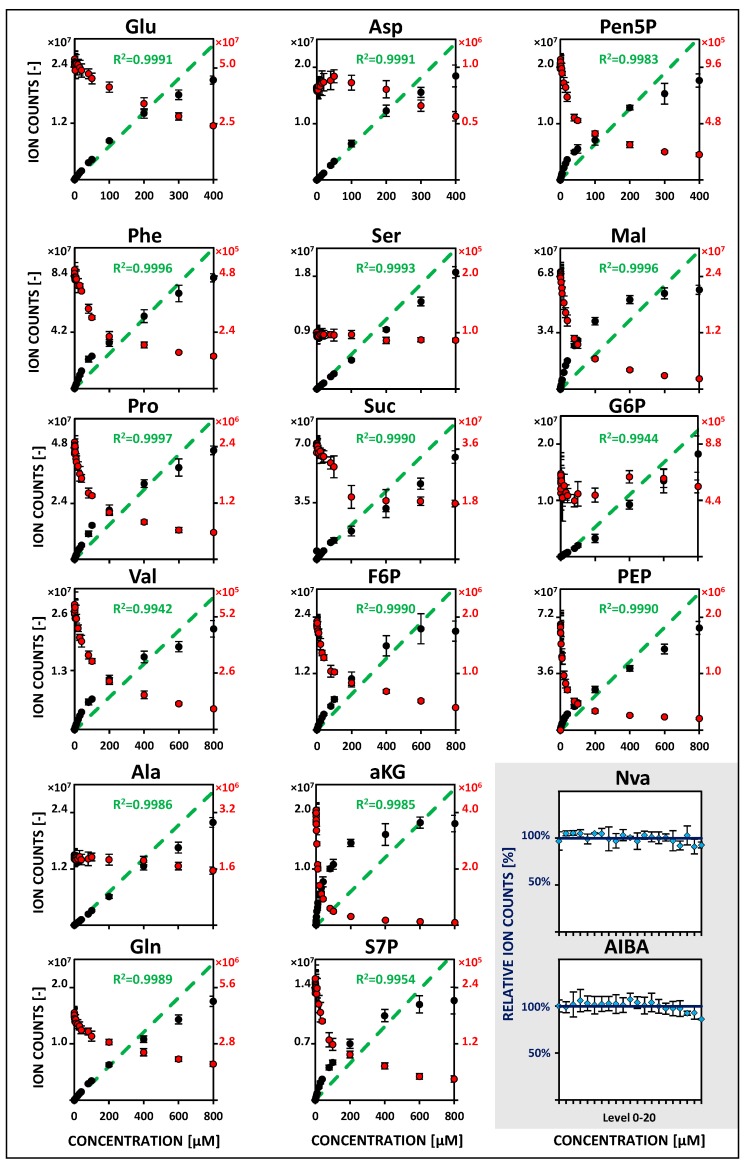
HILIC-based QTOF-HRMS studies in EDR mode of metabolite-specific signal responsivities using U^13^C isotope dilution (IDMS). See Table S6 for related regression parameters. See Figure 1. See Tables S1–S4 for the full names of abbreviated metabolites.

Obtained metabolite-specific linearities vary significantly at low concentrations (5–10 nM to 1 µM) between both MS platforms. Lower linearity limits were determined as lowest significant spiking levels with adequate regression and precision (<20% relative standard deviation, RSD). Within QQQ analysis, most metabolites showed a linearity range between 10–50 nM as lower boundary and 1 µM as the upper limit—except for serine, succinate, and valine with increased lower boundaries (100–200 nM). In contrast to this, less than half of the compounds fulfilled the same criterion with QTOF detection. Extending the calibration range up to 800 µM (for Glu, Asp and Pen5P up to 400 µM), the linearity of instrument responses were revealed to be specific for each compound, sample matrix, and mobile phase elution condition. About half of the metabolites showed already high upper linearity limits of 400–800 µM using the QQQ instrument. Remarkably, almost the same results could be achieved with QTOF detection. In order to increase the significance of the measurements, non-labeled internal standards (50 µM Nva and AIBA) were additionally considered for monitoring instrumental variabilities. Relative deviations from the mean value of corresponding measurements were below 5 and 10% for QQQ and QTOF analysis, respectively. Metabolite-specific linearity ranges and associated relative signal responsivities of internal standards for both MS instruments are summarized in Figure 1 and Figure 2. Corresponding metabolite-specific sensitivities (slope of the extended range) differ considerably between applied platforms and independently optimized operation modes. QQQ analysis exhibits strongly enhanced MRM responsivities in positive ionization mode (ESI+) (by a factor between 2 to 10) compared to QTOF-MS detection. Within negative MRM mode (ESI-), however, corresponding QQQ signal sensitivities are comparatively reduced on average by half. An overview of lower and extended metabolite-specific linearity ranges and related calibration coefficients for both MS platforms is shown in Table 1 and Table 2. 

### 2.3. Instrumental Precision, Retention Time Stabilities, and Methodical Detection Limits

As expected for MRM mode, the QQQ-MS/MS platform provided the best performance in terms of precision, by far. Mean values of RSDs were consistently below 5%. In the analyzed dynamic range, most metabolites exhibited significantly diminished RSDs (about 50%) with increased concentration levels (>100 µM). On the contrary, QTOF-HRMS detection revealed larger but acceptable deviations between 5 and 20% for all metabolites, irrespective of measured concentration levels. Strikingly, no statistically significant correlation could be found between applied levels and RSDs as confirmed by regression analysis and one-way ANOVA (data not shown). 

HILIC-based retention time stabilities were considerably high. Obtained absolute standard deviations of reference standards range between 0.01–0.10 and 0.01–0.07 min for QQQ and QTOF analysis, respectively. Analyzed metabolites were sufficiently retained compared to the void volume and interfering non-polar compounds (>3 × t_0_). Absolute values, however, are significantly shifted between both LC-MS platforms due to the different pump systems and configurations (Table 1 and Table 2).

Methodical detection limits (MDL) were calculated as the amount of compound needed to create statistically significant peak areas distinguishing from the background noise. MDLs (sample concentration) were based on the standard deviations of quadruplicates (*n*−1 = 3 degrees of freedom) at the lower linearity boundary multiplied by the expansion coefficient (*t* = 4.541) for defining the 99% confidence level [28]. In-column detection limits (IDL) (injected amount of substance) ranged between 6.8–304.7 and 28.7–881.5 fmol for QQQ and QTOF analysis, respectively. Accordingly, median values of related MDLs were approximately 12 nM and 56 nM. Using QTOF-HRMS detection only three metabolites (aKG, Mal and Gln) showed MDLs below 20 nM (100 fmol on column). By contrast, more than 80% of the analyzed metabolites fulfilled the same criterion with QQQ-MRM detection, reflecting the lower linearity limits and enhanced precision of the platform. Lowest MDLs were achieved for alanine (1.37 nM), aspartate (3.06 nM), and phenylalanine (3.47 nM). An overview of the metabolite specific MDLs for both MS platforms is shown in Table 1 and Table 2.

### 2.4. Isotope Dilution Mass Spectrometry (IDMS) 

The use of isotope dilution mass spectrometry (IDMS) as quantification strategy for QQQ (MRM mode) and QTOF (MS mode) analysis was comparatively studied by constant addition of U^13^C-metabolite extracts to non-labeled calibration standard mixtures. Cellular extracts were prepared from isotopically stationary labeled *C. glutamicum* U^13^C-batch cultures in the late exponential growth phase (U^13^C-d-glucose as sole carbon source). The quality of the internal standard solutions was evaluated by measurement of blank samples without further addition of non-labeled metabolite standards (Level 0). The isotopic purity of the fully labeled extracts was notably high. Regardless of the applied MS platform, metabolite-specific relative abundance ratios of non-labeled compounds (^12^C/U^13^C) were consistently below 2.0% with a median of approximately 1%, except for serine (Table 3). 

Non-labeled analyte peak areas of simultaneously measured calibration levels were normalized by areas of U^13^C-isotopologues as internal standards. Compared to non-normalized calibration curves linearity ranges are significantly extended with sufficient regression (*R*^2^ > 0.99), coinciding with decreasing responsivities of corresponding U^13^C-analogs (Figure 1 and Figure 2). For both instruments, all metabolites showed constant lower boundaries and upper linearity limits at maximum concentrations (up to 800 µM) enabling accurate quantifications over a broad dynamic range (Table 1 and Table 2). Additional error contributions of detected U^13^C-responsivities had only a negligible effect on measuring precision. Mean values of RSDs were comparable between 5 and 20% for QQQ and QTOF analysis, respectively. Consequently, IDLs were only slightly affected with ranges between 13.6–329.6 and 63.3–750.5 fmol on column with MDL median values of approximately 7 and 32 nM for QQQ and QTOF analysis, respectively. Lowest MDLs were achieved for aspartate (2.72 nM), phenylalanine (2.81 nM), and proline (4.32 nM) using QQQ-MRM detection (Table 1 and Table 2).

Fully labeled *C. glutamicum* extracts were finally evaluated with respect to concentration profiles of selected metabolites. The normalized calibration curves enabled a precise quantification of the applied U^13^C levels by internal calibration. As depicted in Table 3, sample analysis revealed a high similarity between quantitative results using QQQ and QTOF analysis confirming the applicability and transferability of the approach. Obtained absolute values only deviated by 1–9%. Notably, the resulting U^13^C-concentration profiles varied from µM to mM range mimicking native intracellular pool size distributions and could be precisely adjusted to targeted metabolites and concentration levels. 

### 2.5. Metabolite-Specific Spectral Accuracies 

**Figure 3:**
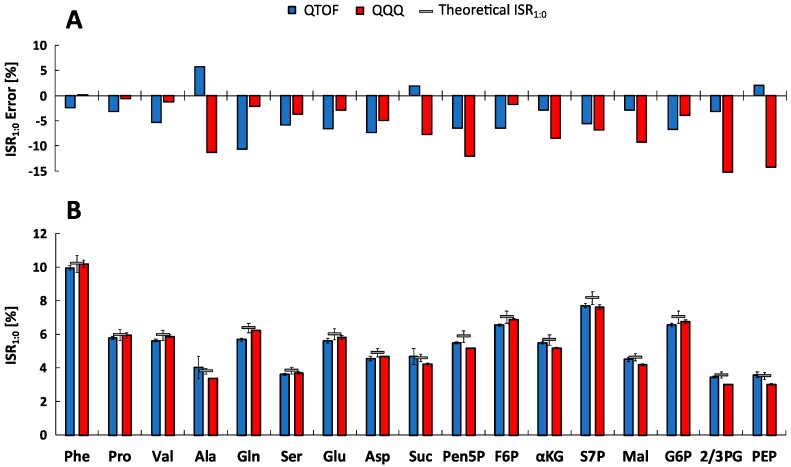
Evaluation of *m+1*/*m+0* Isotopologue Ratios (ISR_1:0_). (**A**) Experimental metabolite-specific mean ISR_1:0_ values (*n* = 5) determined by the QTOF-HRMS (blue) and QQQ-SIM (red) systems in defined non-labeled mixtures of reference standards and theoretical calculated ISR_1:0_ (grey) [29]. (**B**) Corresponding relative ISR_1:0_ errors of the experimental compared to the theoretical ratio for both systems. See Tables S1–S4 for the full names of abbreviated metabolites.

Defined non-labeled metabolite mixtures of reference standards were analyzed with both LC-MS systems to investigate spectral accuracy. To evaluate experimental data, theoretical abundances of naturally occurring isotopologues and related uncertainties were calculated following the approach of Somoano-Blanco et al., 2016 [29]. As a measure of spectral accuracy experimental metabolite-specific *m+1*-to-*m+0* isotope ratios (ISR_1:0_) were calculated and compared with theoretical values (Figure 3). 

**Figure 4:**
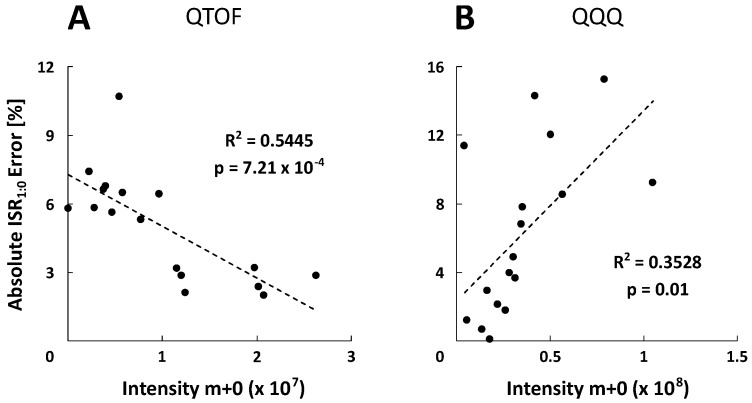
Correlation between absolute ISR_1:0_ error values and intensities of the monoisotopic ion (*m+0*). Each data point corresponds to one metabolite of a non-labeled reference standard mix. Linear regression coefficients (*R*^2^) and p-values are depicted for the QTOF‑HRMS (**A**) and QQQ-SIM detection (**B**).

The experimental ISR_1:0_ mean standard deviations of 3.21 ± 4.00% and 1.26 ± 0.66% were obtained for all selected metabolites and five measurements (*n* = 5) using the QTOF- (MS mode) and QQQ-system (SIM mode), respectively. Based on the variability of the natural isotopic background, theoretical ISR_1:0_ showed similar standard deviations about 5%. For further investigation, error calculations were performed resulting in relative ISR_1:0_ errors. Figure 3A shows that the majority of metabolite-specific ISR_1:0_ errors are negative, illustrating a systematic underestimation of the experimental isotope ratios. However, the average absolute ISR_1:0_ error value of all metabolites is in the range of the recommended 5% isotope abundance error [22] with 5.03 ± 2.30 % for QTOF and 6.26 ± 4.72 % for QQQ analysis, confirming a sufficient spectral accuracy of both MS platforms. Since the individual ISR_1:0_ error profiles of metabolites differ significantly between QTOF and QQQ detection, an in-depth analysis was performed to identify data correlations. Remarkably, both systems exhibit an opposed roughly linear correlation between ISR_1:0_ errors and signal intensities of corresponding monoisotopic ions (*m+0*) (Figure 4). The QTOF system showed decreased ISR_1:0_ errors at higher intensities (*R*^2^ = 0.5445, *p* < 7.21 × 10^−4^) and the QQQ system exhibited decreased ISR_1:0_ errors at lower intensities of the *m+0* ions (*R*^2^ = 0.3528, *p* < 0.01), pointing towards different underlying mechanisms of uncertainty. 

### 2.6. Isotopologue Analysis in Biological Sample Matrices

Next, spectral accuracies were comparatively assessed in complex biological sample matrices. For this purpose, a *C. glutamicum* culture was continuously cultivated and isotopically labeled using a tracer feed with 67% [U^13^C]-d-glucose and 33% [1-^13^C_1_]-d-glucose. Samples were taken directly before (t_0_) as well as 28 sec (t_1_), 52 sec (t_2_), and 10 h (t_3_) after tracer addition. Isotope mass distributions of resulting metabolite extracts were analogously analyzed (*n* = 5) with both MS systems, omitting further correction of the isotopic distributions regarding the natural abundance of isotopes, due to the assessment of spectral accuracy. Measurements of the non-labeled sample extract (t_0_) were evaluated for spectral accuracies by calculation of experimental ISR_1:0_ values. Experimental ISR_1:0_ mean standard deviations of 3.89 ± 3.54% (QTOF-HRMS) and 4.01 ± 3.01% (QQQ-SIM) were in the range of the recommended 5% isotope abundance error [22] and comparable to results in defined aqueous standard mixtures. 

**Figure 5:**
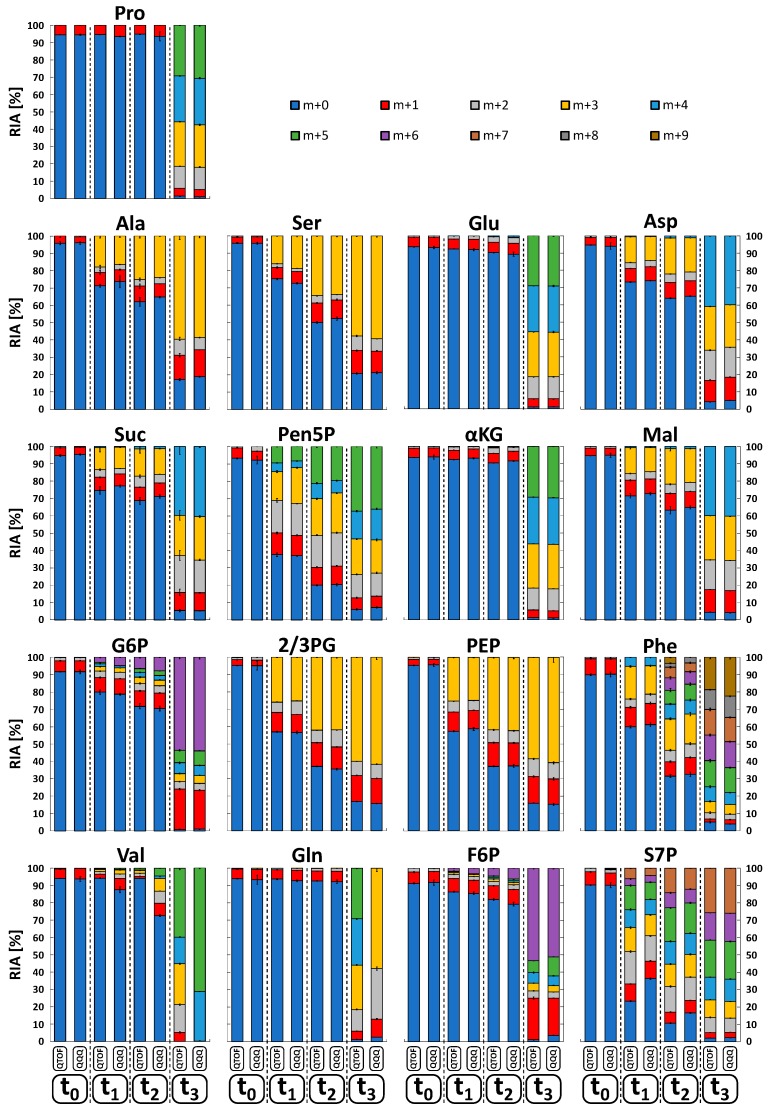
Relative isotopologue abundances (RIA) of selected metabolites. Based on dynamically ^13^C-labeled *C. glutamicum* metabolite extracts (t_0_ < 0 s, t_1_ = 28 s, t_2_ = 52 s, t_3_ = 10 h), analyzed by QTOF-HRMS and QQQ-SIM (*n* = 5). See Tables S1–S4 for the full names of abbreviated metabolites.

**Figure 6:**
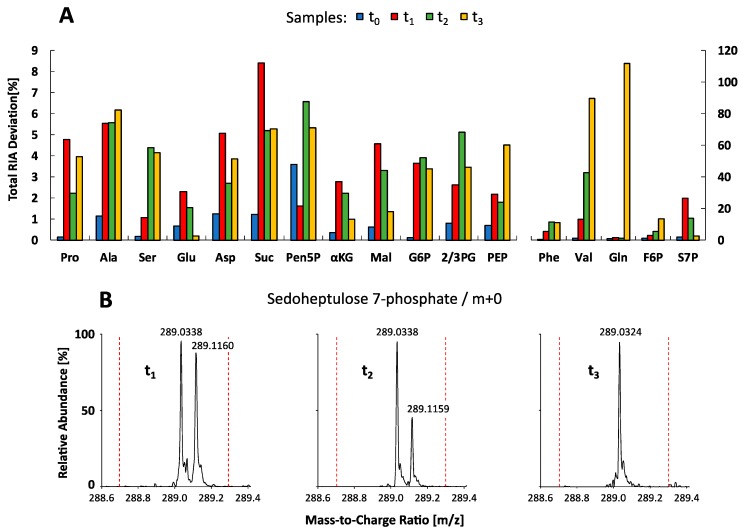
Conformity analysis of relative isotopologue abundances (RIA). (**A**) Total RIA deviations between the QTOF-HRMS and QQQ-SIM ^13^C-labeled datasets (t_0_ < 0 s, t_1_ = 28 s, t_2_ = 52 s, t_3_ = 10 h) are shown as bar plots for all metabolites. See Tables S1–S4 for the full names of abbreviated metabolites. (**B**) High-resolution QTOF mass spectra (t_R_ = 22.4 min) of ^13^C-labeled *C. glutamicum* metabolite extracts (t_1_–t_3_) showing the monoisotopic ion cluster of sedoheptulose 7-phosphate (*m/z* 289.0338 and *m/z* 289.0324) and an adjacent ion cluster identified as argininosuccinic acid (*m/z* 289.1160 and *m/z* 289.1159). Dashed red lines indicate the mass resolution of the QQQ-SIM system (*m/z* 0.3).

To investigate the dynamic labeling of selected intracellular metabolite pools, corresponding relative isotopologue abundances (RIA) in all sample extracts (t_0_–t_3_) were comparatively measured and visualized as bar plots in Figure 5. Labeling analysis revealed a consistent ^13^C-labeling propagation up to the isotopic steady state, demonstrating the quality of the tracer experiment for benchmarking of the LC-QTOF and LC-QQQ mass detection in complex endogenous extracts. The related precision of both instruments was evaluated by calculating the mean standard deviation of all RIAs for all metabolites in each sample. Similar to the analysis of reference standards, the LC-QQQ system (SIM mode) exhibited higher precision with a mean deviation of 2.47 ± 5.58 %, compared to the LC-QTOF system (MS mode) with 4.87 ± 6.37 %. Conformity of the data sets was evaluated by calculating total RIA deviations for each compound, comparing major variations and trends between the measurements. Figure 6A shows two distinct groups of metabolites which significantly differ in the amount of total RIA deviations.

The group on the left of Figure 6A depicts deviations below 10% between both MS systems which basically indicates a fairly good accordance between both data sets not giving rise to any specific trend of deviation. On the right side, five metabolites (Phe, Val, Gln, F6P, and S7P) show notably higher total RIA deviations than 10%. As an example, the RIA of the monoisotopic ion (*m+0*) of S7P in sample t_1_ and t_2_ is noticeably increased by applying QQQ-SIM detection compared to QTOF-HRMS analysis (Figure 5). In-depth analysis of the QTOF high-resolution data set was conducted by investigation of MS spectra focusing on the accurate S7P monoisotopic mass (*m+0*) in negative polarity mode (*m/z* 289.0330). All samples showed distinct ion clusters for S7P between *m/z* 289.0324 and *m/z* 289.0338. However, in samples t_1_ and t_2_, an adjacent ion cluster at *m/z* 289.1160 and *m/z* 289.1159 correlated with the high discrepancy in total RIA deviations (Figure 6B). This ion cluster could be identified as argininosuccinic acid (C_10_H_18_N_4_O_6_) via accurate mass (+2.07 ppm), natural isotope distribution and similar dynamic labeling information compared to aspartate, which is a direct precursor in the arginine biosynthesis. In contrast to QTOF-HRMS analysis with high mass accuracy (*m/z* 0.003) the limited mass resolution of QQQ-SIM detection (*m/z* 0.3, red dashed line in Figure 6B) leads to an overestimation of the monoisotopic S7P mass. Similar mass interferences could be observed for valine (*m+0* to *m+3*) because of co-elution and overlapping isotopologue spaces with proline (*m+2* to *m+5*) (Figure 6A). Consequently, LC-QQQ detection was not able to distinguish between these isotopologues with a small difference of only *m/z* 0.009. On contrast, LC-QTOF allowed to identify all valine and proline isotopologues with an extraction window of 10 ppm (*m/z* ∼0.001).

## 3. Discussion

MS-based absolute quantification of isotopically labeled metabolites is usually performed on QQQ instruments due to their excellent sensitivities and extended linear dynamic ranges [30,31]. Even though recent instrumental improvements of ion sources and detectors have led to increased use of TOF or QTOF hybrid platforms for quantitative LC-MS detection, there is still a lack of cross-platform metabolic studies [24,25,32]. In this work, we performed a systematic and comparative study for the quantification of metabolite pools and determination of isotopologue distributions of a representative cross-section of intracellular compounds using absolute and relative LC-QQQ and -QTOF analysis. The methodical validation for the quantification of non-labeled compounds leads to comparable linearity ranges up to 3–5 orders of magnitude, varying metabolite-specific signal sensitivities, and a significantly enhanced measuring precision using QQQ-MRM detection. In consequence, QTOF-HRMS detection limits are significantly increased but are generally sufficient for reliable and direct quantification of quenched and extracted intracellular pool concentrations of common metabolites. For both platforms, achieved IDL ranges are at least equal or exceed metabolite-specific detection limits of comparable LC-ESI-MS approaches [13,14,33]. However, the reliable quantification of absolute concentrations with highest precision requires the adaption of equidistant and weighted calibration levels. 

In contrast to reversed-phase liquid chromatography (RPLC), HILIC is characterized by the analyte partition between a partially immobilized water-enriched layer and a dynamic polar-organic mobile phase [34,35]. Using zwitterionic stationary phases (ZIC HILIC) retention is additionally influenced by electrostatic (ionic) interactions when applied buffer concentrations are below 20 mM [36]. In sum, optimized conditions enable the adequate separation of polar compounds, covering a great number of key metabolites of major microbial pathways with high selectivity in a few runs, without prior derivatization or interfering ion pair agents [13]. The observed retention time stabilities of targeted metabolites allowed the application of highly customized measuring programs which increased corresponding signal sensitivities by optimized MS detection settings and polarity modes (Tables S1–S4). 

Electrospray ionization is a competitive process, resulting in changing signal responsivities of selected analytes by co-eluting compounds in varying sample matrices [37,38]. As depicted in Figure 1 and Figure 2, limited electrospray droplet surfaces in applied classical (QQQ) or JetStream (QTOF) ESI interfaces also provoked inherently restricted ionization efficiencies and non-linear calibration curves at higher concentration [39]. Accordingly, the pronounced matrix effects in varying multicomponent cellular extracts have led to the almost obligatory application of IDMS within ESI-MS-based quantitative metabolomics [40,41,42]. The application of U^13^C-metabolites as internal standard compensates non-wanted impacts of metabolite degradation and ion suppression and enables an accurate determination of absolute metabolite concentrations by time-saving external calibration. However, related U^13^C-isotopologue standards can be expensive or are still unavailable for most microbial metabolites. Commercially available uniformly labeled biomasses (U^13^C-lyophilized algal cells) provide a convenient and cost-effective alternative for a broad range of common intracellular metabolites [13,43]. However, resulting cellular extracts often differ strongly in the concentration of targeted metabolites or provoke additional ion suppression by an undesired addition of interfering non-targeted compounds. Tailor-made cultivations of microorganisms on fully labeled ^13^C-carbon sources represent an excellent, but often somewhat laborious, alternative to obtain high-quality extracts [40]. Instead, we present a straightforward and easily transferable preparation strategy using *C. glutamicum* U^13^C-shake flask cultures of the late exponential growth phase. The quality and applicability of obtained internal standard solutions (Table 3) was independently confirmed and validated by QQQ and QTOF analysis and allowed a precise adjustment to concentration profiles of targeted microorganisms. IDMS normalization only slightly affected determined detection limits and measuring precision (Table 1 and Table 2) and enabled the accurate quantification in multiple sample matrices with pronounced concentration dynamics by strongly extended linear dynamic ranges (Figure 1 and Figure 2).

The reliable identification of intermediates and isotope labeling patterns in complex biological matrices within metabolic tracer studies (e.g., ^13^C) requires comparatively high or at least sufficient mass accuracy of time-resolved data. The straightforward evaluation of spectral accuracy was based on the comparison of the *m+1*-to-*m+0* isotopologue abundance ratios (ISR_1:0_) [44,45,46] of obtained experimental MS datasets and theoretical, natural isotope distributions [29]. Experimental ISR_1:0_ mean standard deviations of measured metabolite standard mixtures were in the range of the recommended 5% isotope abundance error [22] and previously published datasets for QTOF [47] and QQQ platforms [48]. 

However, Figure 3 reveals a systematic underestimation of ISR_1:0_ values with both MS platforms, which was previously also described for TOF instruments [21,46]. Related reports assume an unexpected non-linearity of the detector at low counts which underrates low abundance masses [46] contradicting the common understanding of detector saturation. Similar effects become evident for QTOF analysis with increased metabolite-specific absolute ISR_1:0_ error values at lower abundances of the corresponding monoisotopic ion (*m+0*) (Figure 4). Consequently, the latter mirrors higher detection limits due to comparatively lowered measuring precision (Table 2). On the contrary, QQQ analysis unraveled opposite trends showing increased errors with higher ion abundance but at least one order of magnitude higher signal responsivities of SIM detection. This inverse relationship of absolute ISR_1:0_ errors might be due to the different detection modes used for QQQ (SIM) and QTOF (full scan) (Figure 4).

Challenging the applicability of the approach for isotopic tracer experiments both LC-MS platforms were further investigated in non- and ^13^C-labeled intracellular *C. glutamicum* metabolite extracts. The evaluation of natural isotopic backgrounds (non-labeled extracts) yielded a similar outcome as reference standard mixtures of high purity, indicating a sufficient spectral accuracy for both LC-MS systems even in complex biological matrices. However, other cellular extracts or targeted metabolites require related validation steps to exclude interferences by isobaric compounds with same retention time. The achieved accuracy is comparable with other studies like the GC-MS analysis of amino acid isotopomers from total *Escherichia coli* biomass hydrolyzates which resulted in an absolute mean ISR_1:0_ error of 4.66 ± 4.70% [49] or the LC-MS/MS-based determination of ^13^C-patterns for central carbon metabolites of *Bacillus subtilis* with a mean error of 8.28 ± 9.93% [30]. 

Even though certified isotopic reference material exists in elemental mass spectrometry [50,51] there is no reference material certified for molecular isotope distributions. By analogy, no theoretical data or reference material was available for ^13^C-labeled *C. glutamicum* metabolite extracts, enabling the evaluation of spectral accuracy in these specific biological matrices. Hence, conformity analysis was performed and obtained RIAs lead to sufficient measurement precision and agreement between both MS datasets for most of the metabolites (Figure 6A, left side). However, a minor group of metabolite RIAs differed significantly. The main reasons were identified as co-eluting, almost isobaric isotopologues and the limited mass resolution of QQQ-SIM detection (Figure 6A, right side). Corresponding ion cluster interferences in complex sample matrices are well known as an inherent problem of QQQ or Q platforms due to the limited resolution width of the applied quadrupole mass analyzers [52]. In such cases, only high-resolution QTOF analysis enables the identification and data extraction of interfering compounds but is still limited in the separation of co-eluting constitutional isomers. 

The successful chromatographic separation of isobaric compound pairs—such as fructose-/glucose 6-phosphate, citrate/iso-citrate, and leucine/isoleucine [13]—which are frequently present in intracellular extracts, confirms the quality of the applied alkaline HILIC method. Consequently, mass interferences could be minimized leading to precise and unbiased RIA accuracies, especially for QQQ instruments used in the more sensitive but less selective SIM mode. Furthermore, selectivity can be significantly increased by combined MS/MS analysis of parent and fragment isotopes considering the reaction stoichiometry of selected networks [31,53]. Such approaches provide additional positional labeling information, however at the expense of metabolite coverage, for example limiting analysis on phosphorylated compounds [54]. In addition, even targeted analyses of single metabolites such as aspartate may already lead to 47 labeling measurements with multiple fragmentations to quantify the complete isotopomer space [12].

In sum, systematic evaluation of QQQ-SIM isotopologue analysis demonstrated sufficient spectral accuracy, capable of extracting similar RIAs for 70 % of the focused polar metabolites in endogenous *C. glutamicum* extracts, due to the excellent performance of HILIC chromatography. However, only QTOF-HRMS ensured the comprehensive and valid analysis of the total isotopologue space of focused compounds with ultimate spectral accuracy.

## 4. Conclusions

The experimental and analytical approach outlined here facilitates a systematic cross-platform study for the comprehensive analysis of demanding non- and ^13^C labeled intracellular extracts using HILIC-based QQQ and QTOF detection. Both approaches are well suited for the accurate quantification of pool concentrations and isotope distributions of a broad range of common intracellular key metabolites with sufficient precision and spectral accuracy, resulting in high-quality data sets for ^13^C-tracer based flux analysis. 

QQQ platforms are particularly appropriate for quantitative profiling of a clearly defined group of metabolite pools with highest precision. Further exploiting the approach, selective MS/MS ^13^C fragmentation studies, moreover, enable in depth analysis of highly branched networks with known stoichiometry providing additional information by targeting of distinct positional labeling/transition states. In turn, QTOF analysis is predestined for the comprehensive and interference-free analysis of global reaction networks, allowing simultaneous and time-saving detection of several dozens of metabolites with acceptable precision in one single run. Furthermore, superior mass resolution enables potential ^13^C analyses of only partially elucidated pathways including the de novo identification of so far unknown metabolic routes and intermediates by analysis of the entire isotopic space. 

## 5. Material and Methods

### 5.1. Analytical Chemicals

MS-grade water and acetonitrile for the preparation of mobile phases and samples were purchased from Carl Roth (Karlsruhe, Germany). Metabolite standards and reagents (>99% p.a) were purchased from Sigma-Aldrich (Schnelldorf, Germany). The standard stock solutions were prepared in MS-grade water and stored at −70 °C. [U^13^C]-d-glucose (99 atom%) for the preparation of fully labeled cellular extract for IDMS was purchased from Silantes (München, Germany). [U^13^C]- (99 atom%) and [1-^13^C_1_]-d-glucose (99 atom%) for isotopic tracer experiments were purchased from Cambridge Isotope Laboratories (Tewksbury, USA) and Sigma-Aldrich (Schnelldorf, Germany), respectively. Chloroform (>99% p.a.), HPLC-grade water, and methanol for quenching and extraction was purchased from Carl Roth (Karlsruhe, Germany) and VWR chemicals (Darmstadt, Germany), respectively.

### 5.2. Strain and Seed Train

The *C. glutamicum* ATCC 13032 wild-type was obtained from the American Type Culture Collection. Cryogenic cultures (25% (*v/v*) glycerol at −70 °C) were spread on 2 × TY [55] agar plates. After incubation for (48–60 h) at 30 °C, colonies were used to inoculate 5 mL of 2 × TY complex medium filled in glass reaction tubes. After 8 h initial pre-cultures were inoculated in 50 mL CGXII medium [56] supplemented with 40 g L^−1^ [U^13^C]-d-glucose as the sole carbon source and 21 g L^−1^ MOPS buffer (adjusted to pH 7.0). After 13 h unlimited batch growth adapted biomasses were harvested by centrifugation for 10 min at 4000× *g* and 4 °C (Eppendorf 5403, Hamburg, Germany) and resuspended in isotonic 0.9% (*w/v*) sodium chloride solution. All shake flask cultivations were performed under ambient pressure at 30 °C in 500 mL baffled shake-flasks on a rotary shaker (Infors HAT CH-4203, Einsbach, Germany) at 120 rpm.

### 5.3. Preparation of Fully Labeled ^13^C-Metabolite Extracts from *C. glutamicum*

Prepared pre-cultures were inoculated (0.2 g_CDW_ L^−1^) in 50 mL CGXII medium supplemented with 40 g L^−1^ [U-^13^C]- d-glucose as sole carbon source and 21 g L^−1^ MOPS buffer (adjusted to pH 7.0) in 500 mL baffled shake-flasks. Stationary labeled cultures were pooled (150 mL) in the late exponential phase (µ_max_ 0.33 h^−1^ ± 0.003) at a biomass concentration of 12 g_CDW_ L^−1^ after 12.5 h unlimited batch growth. Biomasses were harvested by centrifugation (5 min, 3000× *g*, 4 °C) and washed with isotonic 0.9% (*w/v*) sodium chloride solution (5 min, 3000× *g*, 4 °C). Preheated water (100 °C) was added aiming for an extraction concentration of 50 g_CDW_ L^−1^. Obtained suspensions were incubated three times at 100 °C for 2 min in a water bath (Lauda RK20, Lauda, Germany) and resuspended by short-time vortexing. Resulting extracts were chilled on ice water and separated from cell debris by centrifugation (10 min, 20,000× *g*). Aliquots were stored at −70 °C until measurement. 

### 5.4. ^13.^ C-labeling Experiments with *C. glutamicum* in Continuous Culture 

The isotopic tracer experiment with *C. glutamicum* was performed in 3 L stirring tank reactor (KLF 2000 Bioengineering, Switzerland) equipped with a process control system (LabVIEW 2010, National Labs) at 30 °C in continuous fermentation mode. pH was measured with a conventional pH probe (Mettler-Toledo, Germany) and maintained at 7.4 with 25% (*v/v*) ammonium hydroxide. Dissolved oxygen (DO) was monitored by an amperometric pO_2_ electrode (Mettler-Toledo, Germany) and controlled (>30%) by adjusting the stirrer speed (250 to 600 min^−1^) and aeration rate (0.12 to 0.8 L min^−1^). Oxygen and carbon dioxide contents in the exhaust gas were measured by a sensor (BCP-O2/CO2, BlueSens, Germany). Prepared pre-cultures were inoculated (0.3 g_CDW_ L^−1^) in 1.2 L CGXII medium supplemented with 12 g L^−1^ d-glucose as the sole carbon source. After 7.45 h of unlimited batch growth (µ = 0.42 h^−1^) added d-glucose was depleted entirely reaching a biomass concentration of 6 g_CDW_ L^−1^. The continuous mode was started by feeding fresh supplemented CGXII medium with a dilution rate of 0.4 h^−1^ keeping a working volume of 1.2 L. After 12.5 h (five residence times) online measurements (constant OUR and CER) indicated that the culture was at metabolic steady state. The reactor was switched to labeled CGXII feed containing 67% [U-^13^C]-d-glucose and 33% [1-^13^C_1_]-d-glucose. After another 10 h (four residence times) the culture was expected to have reached isotopic stationarity. Samples for intracellular metabolome analyses were generated according to an adapted sequential protocol of Koning and Dam, 1992 [57] via cold-methanol quenching (CMQ) and methanol-chloroform extraction (CME). For rapid quenching of the cellular metabolism 2 mL withdrawn biosuspension was directly mixed with 3 mL 60% (*v/v*) aqueous methanol quenching solution precooled to −60 °C and centrifuged for 10 min at 4000 *g* and −1 °C (rotor precooled to −20 °C). Residual biomasses were frozen in liquid nitrogen and temporarily stored at −70 °C. Subsequently, 1 mL precooled (−20 °C) 50% (*v/v*) aqueous methanol extraction solution was added. Cell pellets were resuspended by short-time vortexing and mixed with 1 mL precooled (−20 °C) chloroform. Resulting suspensions were incubated for 2 h at −20 °C in a rotary overhead-shaker and centrifuged for 10 min at 4000− *g* and 4 °C. The upper aqueous methanol phase was carefully removed and temporarily stored at −70 °C until measurement. 

### 5.5. Chromatographic Conditions 

QQQ-MS/MS studies were performed on an Agilent 1200 HPLC system consisting of a degasser, a binary pump, a thermostated column compartment and a bio-inert autosampler (Agilent 1260), maintained at 5 °C. QTOF-HRMS studies were performed on a comparable bio-inert Agilent 1260 HPLC system with a quaternary pump system. Both systems used the same LC-MS method for quantification of non-derivatized metabolites, which was based on a bicratic zwitterionic hydrophilic interaction chromatography (ZIC-pHILIC) under alkaline mobile phase conditions [13]. Prepared standard mixes or endogenous cellular extracts (60% acetonitrile (*v/v*) and 10 mM ammonium acetate, pH 9.2) were injected (5 µL) onto a Sequant ZIC-pHILIC column (150 × 2.1 mm, 5 µm, Merck Millipore, Darmstadt, Germany) with guard column (20 × 2.1 mm, 5 µm, Merck Millipore, Darmstadt, Germany) maintained at 40 °C. Mobile phases were composed of aqueous buffer solutions (10 mM ammonium acetate, pH 9.2) with 90% (*v/v*) acetonitrile for eluent A and 10% (*v/v*) acetonitrile for eluent B. Following gradient program with a constant flow rate of 0.2 mL min^−1^ was applied: isocratic hold 0% B for 1 min, linear gradient from 0% B to 75% B for 30 min, linear gradient from 75% B to 100% B for 4 min, isocratic hold 100% B for 5 min, linear gradient from 100% B to 0% B for 10 min, and equilibration to starting conditions by an isocratic hold 0% B for 15 min.

### 5.6. Data Acquisition for QQQ-MS/MS Studies

Data were acquired on an Agilent 6410B Triple-Quad LC-MS system with a classical ESI interface (Agilent Technologies, Santa Clara, CA, USA). ESI parameters were set as follows: a nitrogen gas flow rate of 10 L min^−1^ at 350 °C, capillary voltages of ±4.0 kV for both ionization modes, and a nebulizer pressure of 30 psi. System control, acquisition, and analysis of data were performed by usage of commercial MassHunter B.04.00 software. Multicomponent mixtures of non-labeled metabolite standards and fully labeled ^13^C-cellular extracts were analyzed with high selectivity in MRM mode based on pre-optimized precursor-to-product ion transitions with a mass resolution of 0.1 µ and adapted MS/MS parameters (Table S1, [13]). Endogenous cellular extracts of ^13^C-isotopic tracer experiments were analyzed with enhanced sensitivity in the SIM mode with a mass resolution of 0.3 µ and analog MS parameters (Table S2). The detection dwell time was adjusted to 100 ms for each MRM and SIM transition. Analytes were detected in the negative (ESI-) and positive ionization mode (ESI+) in one (MRM) or two comprehensive HPLC runs (SIM) with suitable measuring time segments and sufficient peak resolution (at least 15–20 run cycles).

### 5.7. Data Acquisition for QTOF-HRMS Studies

Samples were analyzed by an Agilent 6540 Accurate-Mass Q-TOF LC-MS system with ESI JetStream Technology (Agilent Technologies, Santa Clara, CA, USA). ESI and MS parameters for all experiments were set as follows: drying gas flow rate of 8 L min^−1^ with a gas temperature of 325 °C, nebulizer with 40 psi, sheath gas flow rate of 12 L min^−1^ and sheath gas temperature of 350 °C, capillary voltage of 4000 V, and acquisition rate of 1.4 spectra/s. The system was operated in MS mode (scan range: *m/z* 25–1700) and, owing to the high mass accuracy, precursor ions were used for analysis without the use of any fragmentation. During analysis, online mass calibration was ensured by injection of reference masses in positive (*m/z* 121.050873 and *m/z* 922.009798) and in negative mode (*m/z* 119.03632 and *m/z* 980.016375). System control was performed using MassHunter Data Acquisition (B.06.01, Agilent Technologies, Santa Clara, CA, USA). Analysis concerning linearity range and sensitivity was carried out in extended dynamic range mode (2 GHz) with optimized fragmentor parameters and polarity for each metabolite, achieving cycle times below 1.5 sec. Extraction of chromatograms and integration was done in MassHunter Qualitative Analysis (B.07.00, Agilent Technologies, Santa Clara, CA, USA) with metabolite specific extraction windows depending on mass accuracy (Table S3). Relative quantification of isotopologue abundances was done in high-resolution mode (4 GHz) with a fixed fragmentor parameter of 100 V in the negative mode as well as positive mode. Automated data analysis was carried out in MassHunter ProFinder (B.08.00, Agilent Technologies, Santa Clara, CA, USA) with the “Batch Isotopologue Extraction” feature or by manual analysis in MassHunter Qualitative Analysis (B.07.00, Agilent Technologies, Santa Clara, CA, USA) (Table S4).

### 5.8. Methodical Evaluation of ESI-MS-Based Signal Responsivities

Linear dynamic ranges of both HILIC-based platforms (QQQ-MS/MS and QTOF-HRMS) were evaluated concerning the amount of respectively injected analytes. Multicomponent standard solutions were obtained of defined non-labeled metabolite mixtures and constant addition of 50 µM Nva, AIBA, and KDPG as global internal standards. Isotope dilution (IDMS) was performed by constant addition of U^13^C-labeled *C. glutamicum* extracts (10-fold dilution). Formulations were freshly prepared (every 24 h) from previously at −70 °C stored aliquots. Samples were continuously analyzed in repeated measuring sequences. Focusing on significantly extended concentration ranges and depending on sensitivities of measured metabolites, a calibration range of 10 nM to 800 µM or 5 nM to 400 µM (for Glu, Asp and Pen5P) based on 20 levels was applied. Calibration curves were prepared by linear regression of non-weighted analyte peak areas against the concentration levels of the respective analyte. For isotope dilution, the non-labeled analyte peak areas were additionally normalized by areas of analogs U^13^C-isotopologues as internal standards. 

### 5.9. Relative Quantification of Isotopic Labeling Dynamics 

Natural ^13^C-isotope distribution of metabolite standards and *C. glutamicum* ^13^C-isotopic tracer experiments were comparatively analyzed on the QQQ- and QTOF platform. Time-variant endogenous metabolome extracts or defined non-labeled metabolite mixtures (50 µM) were mixed with 50 µM Nva, AIBA, and KDPG as global internal standards. Formulations were freshly prepared (every 24 h) from previously at −70 °C stored aliquots and were continuously analyzed in repeated measuring sequences. For the analysis of the spectral accuracy of both systems, the isotopologue *m+1*-to-*m+0* ratio (ISR_1:0_) was calculated as

(1) $$ISR_{1:0}\left[\%\right]=\frac{Intensity\;m+1}{Intensity\;m+0}\times 100$$

Theoretical *m+1*-to-*m+0* ratios were calculated according to Somoano-Blanco et al., 2016 [29] to evaluate experimental *m+1*-to-*m+0* ratios. With these values, relative error calculations were performed.

(2) $$ISR_{1:0}\;error\left[\%\right]=\frac{ISR_{1:0}exp-ISR_{1:0}theo}{ISR_{1:0}theo}\times 100$$

To analyze ^13^C-labeled *C. glutamicum* metabolite samples, the relative isotopologue abundance (RIA) of each isotopologue for each measured metabolite and time point was calculated according to

(3) $$RIA_{m+i}\left[\%\right]=\frac{Intensity\;m+i}{\sum_{j=0}^{n}Intensity\;m+j}\times 100$$

n=amount of carbon atoms,0≤i≥n

For comparison of the QQQ- and QTOF-system, deviations of RIA values for each corresponding isotopologue of one metabolite were calculated and summed up to generate the total RIA deviation:

(4) $$Total\;RIA\;deviation\left[\%\right]=\sum_{j=0}^{n}\left|RIA_{m+j}\left(QTOF\right)-RIA_{m+j}\left(QQQ\right)\right|$$

### 5.10. Statistical Analysis

Statistical data analysis was carried out in Microsoft Excel 2010. Linear regression of non-weighted analyte peak areas concentration levels of analytes was performed by applying least squares approximation. Concentration ranges were considered as linear when the squared correlation coefficient (R2) was better than 0.99 for the average of four measurements (*n* = 4). Lower boundaries were determined as lowest significant spiking levels with adequate regression and precision (<20% relative standard deviation, RSD). Methodical detection limits (MDL) were based on the standard deviations of quadruplicates (*n*−1 = 3 degrees of freedom) at the lower linearity boundary multiplied by the expansion coefficient (*t* = 4.541) for defining the 99% confidence level [28]. Correlation analyses were performed by one-way ANOVA. 

## Figures and Tables

**Table 1:** Quantification parameters of targeted metabolites by standard-based external ^12^C and IDMS U^13^C calibration using QQQ-MRM detection in positive (red) and negative (blue) ionization mode (5 µL sample injection). See Tables S1–S4 for the full names of abbreviated metabolites.

*Metabolite*	*Linearity and Sensitivity*							*Precision and Detection Limit*				*Performance*	
Name	^12^C-Calibration				U^13^C-IDMS			^12^C-Calibration		U^13^C-IDMS		LC-MS Parameters	
Abbr.	Min (nM)	Max (µM)	Slope (Cts/nM)	*R*^2^ (-)	Min (nM)	Max (nM)	*R*^2^ (-)	MV RSD (%)	MDL (nM)	MV RSD (%)	MDL (nM)	Retention t_R_ (min)	ESI Polarity
**Phe**	10	100	1424	0.9975	10	800	0.9954	1.94	3.5	1.94	2.8	10.12 ± 0.04	**[+]**
**Pro**	50	100	1346	0.9938	10	800	0.9948	4.72	15.5	1.75	4.3	12.47 ± 0.01	**[+]**
**Val**	200	100	703	0.9955	200	800	0.9986	1.87	17.3	2.09	35.2	12.66 ± 0.01	**[+]**
**Ala**	50	400	244	0.9905	50	800	0.9991	2.13	1.4	2.34	6.6	15.86 ± 0.01	**[+]**
**Gln**	50	800	144	0.9919	50	800	0.9997	2.96	9.6	1.48	5.0	17.16 ± 0.01	**[+]**
**Ser**	100	600	120	0.9932	100	800	0.9999	2.65	60.9	2.74	64.5	17.86 ± 0.01	**[+]**
**Glu**	50	400	189	0.9983	50	400	0.9999	2.18	12.2	1.54	7.3	19.7 ± 0.01	**[+]**
**Asp**	25	400	101	0.9994	25	400	0.9998	3.02	3.1	2.40	2.7	20.17 ± 0.01	**[+]**
**Suc**	100	800	18	0.9914	100	800	0.9995	4.06	55.1	3.40	65.9	20.81 ± 0.04	**[−]**
Pen5P *	25	20	66	0.9949	25	400	0.9998	2.30	6.3	2.40	6.1	21.4 ± 0.05	**[−]**
**F6P**	50	400	38	0.9907	50	800	0.9940	2.01	5.1	2.36	5.9	21.97 ± 0.03	**[−]**
**αKG**	50	10	35	0.9903	50	800	0.9996	2.14	11.8	2.43	13.4	22.11 ± 0.07	**[−]**
**S7P**	50	600	35	0.9914	50	800	0.9996	3.13	17.4	3.34	12.6	22.34 ± 0.04	**[−]**
**Mal**	50	40	93	0.9946	50	800	0.9990	3.71	19.9	2.70	13.2	22.55 ± 0.08	**[−]**
**G6P**	50	800	30	0.9988	50	800	0.9997	2.59	4.2	2.59	6.8	23.04 ± 0.03	**[−]**
**PEP**	50	4	139	0.9975	50	800	0.9998	1.98	9.7	2.05	12.5	24.72 ± 0.10	**[−]**

* Pen5P = Pooled ribose- and ribulose-5-phosphate.

**Table 2:** Quantification parameters of targeted metabolites by standard-based external ^12^C and IDMS U^13^C calibration using QTOF-HRMS detection after EDR tune in positive (red) and negative (blue) ionization mode (5 µL sample injection). See Tables S1–S4 for the full names of abbreviated metabolites.

*Metabolite*	*Linearity and Sensitivity*							*Precision and Detection Limit*				*Performance*	
Name	^12^C-Calibration				U^13^C-IDMS			^12^C-Calibration		U^13^C-IDMS		LC-MS Parameters	
Abbr.	Min (nM)	Max (µM)	Slope (Cts/nM)	*R*^2^ (-)	Min (nM)	Max (nM)	*R*^2^ (-)	MV RSD (%)	MDL (nM)	MV RSD (%)	MDL (nM)	Retention t_R_ (min)	ESI Polarity
**Phe**	50	40	329	0.9995	50	800	0.9996	5.45	28.1	5.75	41.7	11.95 ± 0.03	**[+]**
**Pro**	50	100	142	0.9943	50	800	0.9997	8.73	39.3	7.17	43.3	13.99 ± 0.02	**[+]**
**Val**	400	40	96	0.9949	400	800	0.9942	6.08	75.5	6.53	49.3	14.15 ± 0.02	**[+]**
**Ala**	50	800	28	0.9967	50	800	0.9986	7.45	33.7	5.50	27.1	16.88 ± 0.02	**[−]**
**Gln**	50	400	28	0.9900	50	800	0.9989	6.18	19.9	4.54	31.1	17.94 ± 0.02	**[−]**
**Ser**	100	800	23	0.9999	100	800	0.9993	9.47	75.2	6.81	88.8	18.52 ± 0.02	**[−]**
**Glu**	50	200	73	0.9922	50	400	0.9991	5.78	22.7	3.71	17.6	19.97 ± 0.01	**[−]**
**Asp**	200	200	62	0.9991	200	400	0.9991	9.27	159.2	4.51	93.5	20.36 ± 0.02	**[−]**
**Suc**	200	800	78	0.9950	200	800	0.9990	8.51	151.7	4.73	150.1	21.05 ± 0.04	**[−]**
Pen5P *	200	15	202	0.9938	200	400	0.9983	6.48	89.3	7.15	105.0	21.49 ± 0.04	**[−]**
**F6P**	200	40	84	0.9923	200	800	0.9990	9.27	176.3	7.35	139.9	21.94 ± 0.04	**[−]**
**αKG**	50	2	876	0.9969	50	800	0.9985	6.68	5.7	7.07	12.7	22.18 ± 0.06	**[−]**
**S7P**	100	30	86	0.9943	100	800	0.9954	8.75	32.4	4.98	23.4	22.30 ± 0.05	**[−]**
**Mal**	50	30	509	0.9940	50	800	0.9996	5.13	13.5	4.00	18.5	22.57 ± 0.06	**[−]**
**G6P**	200	800	23	0.9964	200	800	0.9944	17.70	116.8	11.05	93.2	22.89 ± 0.03	**[−]**
**PEP**	100	10	448	0.9905	100	800	0.9990	7.66	71.8	4.60	31.7	24.54 ± 0.07	**[−]**

* Pen5P = Pooled ribose- and ribulose-5-phosphate.

**Table 3:** Quality of U^13^C-labeled *C. glutamicum* extracts for IDMS using QQQ-MRM and QTOF-HRMS detection. See Tables S1–S4 for the full names of abbreviated metabolites.

Metabolite		^12^C/U^13^C-Isotopic Purity (%)		U^13^C-Concentration (µM)	
Abbr.	Name	QQQ	QTOF	QQQ	QTOF
**Phe**	l-Phenylalanine	1.64 ± 0.09	1.32 ± 0.47	1.18 ± 0.01	1.33 ± 0.07
**Pro**	l-Proline	0.51 ± 0.13	0.35 ± 0.05	9.02 ± 0.16	10.67 ± 1.00
**Val**	l-Valine	<MDL	<MDL	3.82 ± 0.07	3.88 ± 0.37
**Ala**	l-Alanine	0.13 ± 0.01	0.15 ± 0.02	64.85 ± 1.40	62.75 ± 1.28
**Gln**	l-Glutamine	0.03 ± 0.00	0.01 ± 0.01	98.37 ± 1.55	92.09 ± 5.87
**Ser**	l-Serine	5.53 ± 0.49	3.25 ± 4.63	3.93 ± 0.03	4.04 ± 0.10
**Glu**	l-Glutamate	0.03 ± 0.00	0.04 ± 0.01	488.95 ± 7.62	460.00 ± 27.48
**Asp**	l-Aspartate	0.42 ± 0.03	0.08 ± 0.09	13.99 ± 0.18	14.55 ± 0.62
**Suc**	Succinate	<MDL	<MDL	251.74 ± 4.06	240.24 ± 9.44
Pen5P *	Pentose-5-phosphate *	1.37 ± 0.16	2.49 ± 0.24	4.21 ± 0.08	3.96 ± 0.09
**F6P**	d-Fructose-6-phosphate	0.31 ± 0.02	0.80 ± 0.12	13.50 ± 0.28	15.46 ± 0.41
**αKG**	α-Ketoglutarate	0.89 ± 0.05	1.93 ± 0.09	3.71 ± 0.04	4.14 ± 0.17
**S7P**	d-Sedoheptulose 7-phosphate	2.04 ± 0.12	3.57 ± 0.83	2.35 ± 0.02	2.34 ± 0.09
**Mal**	l-Malate	<MDL	<MDL	36.82 ± 0.47	32.74 ± 0.56
**G6P**	d-Glucose-6-phosphate	0.26 ± 0.02	0.27 ± 0.46	24.24 ± 0.37	24.76 ± 3.66
**PEP**	2-Phosphoenolpyruvate	1.42 ± 0.07	2.00 ± 0.24	2.80 ± 0.08	2.75 ± 0.09

* Ribose/ribulose-5-phosphate pooled.

## Supplementary Materials

The following are available online at https://www.mdpi.com/2218-1989/9/4/63/s1, Table S1: QQQ-MS/MS Parameters (MRM Mode) and HILIC Program Settings for Targeted Metabolites and U^13^C-Analogs; Table S2: QTOF-HRMS and Extraction Parameters (MS Mode and EDR Tune) and HILIC Program Settings for Targeted Metabolites and U^13^C-Analogs; Table S3A,B: QQQ-MS/MS Parameters (SIM Mode) and HILIC Program Settings for Targeted Metabolites and Corresponding ^13^C-Isotopologues; Table S4: QTOF-HRMS Parameters (MS Mode and HR Tune) and HILIC Program Settings for Targeted Metabolites and Corresponding ^13^C-Isotopologues; Table S5: Regression parameters of HILIC-QQQ-MRM studies of direct and U13C-normalized (IDMS) metabolite-specific signal responsivities using short (up to 1 µM) and extended calibration ranges (up to 400/800 µM, 5 µL sample injection). Supplementary details of Table 1. See Table 3 and Table S1–S4 for the full names of abbreviated metabolites. Table S6: Regression parameters of HILIC-QTOF-HRMS studies of direct and U13C-normalized (IDMS) metabolite-specific signal responsivities using short (up to 1 µM) and extended calibration ranges (up to 400/800 µM, 5 µL sample injection). Supplementary details of Table 1. See Table 3 and Table S1–S4 for the full names of abbreviated metabolites.
