# Supplementary material for: HILIC-Enabled 13C Metabolomics Strategies: Comparing Quantitative Precision and Spectral Accuracy of QTOF High- and QQQ Low-Resolution Mass Spectrometry
Source: Metabolites. 2019 Apr 2;9(4):63. doi: 10.3390/metabo9040063 (PMC6523712; doi:10.3390/metabo9040063)
Supplement: Supplementary file 1 [file metabolites-09-00063-s001.pdf]

Supplemental

Table S1. QQQ-MS/MS Parameters (MRM Mode) and HILIC Program Settings for Targeted Metabolites and U^{13}C-Analogs.

Metabolite			***MS/MS Parameters (MRM Mode)***							***Program (HILIC)***	***Parameters***	
Abbr.	Name	Mass M_i [u]	Precursor Species	U^{12}C-MRM (*m/z*) [u]		U^{13}C-MRM (*m/z*) [u]		Collis. CE [V]	Fragm. F [V]	Retention t_R [min]	Time Segment	ESI Polarity
**Phe**	L-Phenylalanine	165.1	[M+H]$^+$	166	120	175	128	12	75	10.2	**S1**	[+]
**Nva**	L-Norvaline	117.1	[M+H]$^+$	118	72	na	na	10	75	12.1	**S2**	[+]
**Pro**	Proline	115.1	[M+H]$^+$	116	70	121	74	17	75	12.5		
**Val**	L-Valine	117.1	[M+H]$^+$	118	72	123	76	10	75	12.7		
**AIBA**	α-Aminobutyrate	103.1	[M+H]$^+$	104	58	na	na	10	75	14.5	**S3**	[+]
**Ala**	L-Alanine	89.0	[M+H]$^+$	90	44	93	46	10	70	15.9	**S4**	[+]
**Gln**	L-Glutamine	146.1	[M+H]$^+$	147	84	152	88	18	70	17.2	**S5**	[+]
**Ser**	L-Serine	105.0	[M+H]$^+$	106	60	109	62	10	75	17.9		
**Glu**	L-Glutamate	147.1	[M+H]$^+$	148	84	153	88	17	75	19.7	**S6**	[+]
**Asp**	L-Aspartate	133.0	[M+H]$^+$	134	74	138	76	13	75	20.2		
**Suc**	Succinate	116.0	[M-H]$^-$	117	73	121	76	9	82	20.8	**S7**	[-]
**Pen5P**	Pentose-5-phosphate	230.0	[M-H]$^-$	229	97	265	97	6	90	21.4		
**F6P**	D-Fructose-6-phosphate	260.0	[M-H]$^-$	259	97	265	97	15	100	22.0		
**αKG**	α-Ketoglutarate	146.0	[M-H]$^-$	145	101	150	105	4	70	22.1		
**S7P**	D-Sedoheptulose 7-phosphate	290.0	[M-H]$^-$	289	97	296	97	12	90	22.3		
**Mal**	L-Malate	134.0	[M-H]$^-$	133	115	137	119	7	80	22.5		
**G6P**	D-Glucose-6-phosphate	260.0	[M-H]$^-$	259	97	265	97	12	100	23.0		
**2/3PG**	2/3-Phosphoglycerate	186.0	[M-H]$^-$	185	79	188	79	38	90	23.8	**S8**	[-]
**KDPG**	2-dehydro-3-deoxy-6-phospho-D-gluconate	258.0	[M-H]$^-$	257	97	na	na	8	75	24.5		
**PEP**	2-Phosphoenolpyruvate	168.0	[M-H]$^-$	167	79	170	79	14	70	24.7		

Table S2. QTOF-HRMS and Extraction Parameters (MS Mode and EDR Tune) and HILIC Program Settings for Targeted Metabolites and U13C-Analogs.

Metabolite			***MS and Extraction Parameters (Tune)***					***Program Parameters (HILIC)***		
Abbr.	Name	Mass Mi [u]	Ion Species	U12C-Ion (*m/z*) [u]	U13C-Ion (*m/z*) [u]	Fragm. F [V]	ppm	Retention t_R [min]	Time Segment [min]	ESI Polarity
**Phe**	L-Phenylalanine	165.0790	$[M+H]^+$	166.0863	175.1164	80	10	12.0	**0-15.2**	**[+]**
**Nva**	L-Norvaline	117.0790	$[M+H]^+$	118.0863		80	10	13.7		
**Pro**	Proline	115.0633	$[M+H]^+$	116.0706	121.0874	80	10	14.0		
**Val**	L-Valine	117.1000	$[M+H]^+$	118.0863	123.103	80	10	14.1		
**AIBA**	α-Aminobutyrate	103.1000	$[M-H]^-$	102.0561		100	10	15.7	15.2-20.8	[-]
**Ala**	L-Alanine	89.0477	$[M-H]^-$	88.0404	91.0505	100	10	16.9		
**Gln**	L-Glutamine	146.0691	$[M-H]^-$	145.0619	150.0786	100	10	17.9		
**Ser**	L-Serine	105.0426	$[M-H]^-$	104.0353	107.0454	100	10	18.5		
**Glu**	L-Glutamate	147.0532	$[M-H]^-$	146.0459	151.0627	100	10	20.0		
**Asp**	L-Aspartate	133.0375	$[M-H]^-$	132.0302	136.0437	100	10	20.4		
**Suc**	Succinate	116.0121	$[M-H]^-$	117.0193	121.0328	80	50	21.1	20.8-30	[-]
**Pen5P**	Pentose-5-phosphate	230.0192	$[M-H]^-$	229.0119	234.0287	100	10	21.5		
**F6P**	D-Fructose-6-phosphate	260.0297	$[M-H]^-$	259.0224	265.0426	100	10	21.9		
**αKG**	α-Ketoglutarate	146.0215	$[M-H]^-$	145.0142	150.031	80	10	22.2		
**S7P**	D-Sedoheptulose 7-phosphate	290.0403	$[M-H]^-$	289.033	296.0565	100	10	22.3		
**Mal**	L-Malate	134.0215	$[M-H]^-$	133.0142	137.0277	80	100	22.6		
**G6P**	D-Glucose-6-phosphate	260.0297	$[M-H]^-$	259.0224	265.0426	100	10	22.9		
**2/3PG**	2/3-phosphoglycerate	185.9929	$[M-H]^-$	184.9857	187.9957	100	10	23.8		
**KDPG**	2-dehydro-3-deoxy-6-phospho-D-gluconate	258.0141	$[M-H]^-$	257.0068		100	10	24.2		
**PEP**	2-Phosphoenolpyruvate	167.9824	$[M-H]^-$	166.9751	169.9852	80	10	24.5		

Table S3A. QQQ-MS/MS Parameters (SIM Mode) and HILIC Program Settings for Targeted Metabolites and Corresponding ^{13}C-Isotopologues.

Metabolite			***MS/MS Parameters (SIM Mode)***			***Program Parameters (HILIC)***		
Abbr.	Name	Formula	Ion Species	$^{13}C_n$-SIM Isotopologues (*m/z*) [u]	Fragm. F [V]	Retention t_R [min]	Time Segment	ESI Polarity
**Phe**	L-Phenylalanine	$C_9H_{11}NO_2$	$[M+H]^+$	*165*, **166**, 167, 168, 169,170 171, 172, 173, 174, **175**, *176*	75	10.2	**S1**	**[+]**
**Nva**	L-Norvaline	$C_5H_{11}NO_2$	$[M+H]^+$	118	75	12.1	**S2**	**[+]**
**Pro**	Proline	$C_5H_9NO_2$	$[M+H]^+$	*115*, **116**, 117, 118, 119, 120, **121**, *122*	75	12.5		**[+]**
**AIBA**	α-Aminobutyrate	$C_4H_9NO_2$	$[M+H]^+$	104	75	14.5	**S3**	**[+]**
**Ala**	L-Alanine	$C_3H_7NO_2$	$[M+H]^+$	*89*, **90**, 91, 92, **93**, *94*	70	15.9	**S4**	**[+]**
**Ser**	L-Serine	$C_3H_7NO_3$	$[M+H]^+$	*105*, **106**, 107, 108, **109**, *110*	75	17.9	**S5**	**[+]**
**Glu**	L-Glutamate	$C_5H_9NO_4$	$[M+H]^+$	*147*, **148**, 149, 150, 151, 152, **153**, *154*	75	19.7	**S6**	**[+]**
**Suc**	Succinate	$C_4H_6O_4$	$[M-H]^-$	*116*, **117**, 118, 119, 120, **121**, *122*	82	20.8	**S7**	**[-]**
**F6P**	D-Fructose-6-phosphate	$C_6H_{13}O_9P$	$[M-H]^-$	*258*, **259**, 260, 261, 262, 263, 264, **265**, *266*	100	22.0	**S8**	**[-]**
**S7P**	D-Sedoheptulose 7-phosphate	$C_7H_{15}O_{10}P$	$[M-H]^-$	*288*, **289**, 290, 291, 292, 293, 294, 295, **296**, *297*	90	22.3		**[-]**
**G6P**	D-Glucose-6-phosphate	$C_6H_{13}O_9P$	$[M-H]^-$	*258*, **259**, 260, 261, 262, 263, 264, **265**, *266*	100	23.0	**S9**	**[-]**
**KDPG**	2-dehydro-3-deoxy-6-phospho-D-gluconate	$C_6H_{11}O_9P$	$[M-H]^-$	257	75	24.5	**S10**	**[-]**
**PEP**	2-Phosphoenolpyruvate	$C_3H_5O_6P$	$[M-H]^-$	*166*, **167**, 168, 169, **170**, *171*	70	24.7		**[-]**

Table S3B. QQQ-MS/MS Parameters (SIM Mode) and HILIC Program Settings for Targeted Metabolites and Corresponding ^{13}C-Isotopologues.

Metabolite			***MS/MS Parameters (SIM Mode)***			***Program Parameters (HILIC)***		
Abbr.	Name	Formula	Ion Species	$^{13}C_n$-SIM Isotopologues (m/z) [u]	Fragm. F [V]	Retention t_R [min]	Time Segment	ESI Polarity
**Phe**	L-Phenylalanine	$C_9H_{11}NO_2$	$[M+H]^+$	*165*, **166**, 167, 168, 169, 170, 171, 172, 173, 174, **175**, *176*	75	10.2	**S1**	**[+]**
**Nva**	L-Norvaline	$C_5H_{11}NO_2$	$[M+H]^+$	118	75	12.1	**S2**	**[+]**
**Val**	L-Valine	$C_5H_{11}NO_2$	$[M+H]^+$	*117*, **118**, 119, 120, 121, 122, **123**, *124*	75	12.7		**[+]**
**AIBA**	α-Aminobutyrate	$C_4H_9NO_2$	$[M+H]^+$	104	75	14.5	**S3**	**[+]**
**Ala**	L-Alanine	$C_3H_7NO_2$	$[M+H]^+$	*89*, **90**, 91, 92, **93**, *94*	70	15.9	**S4**	**[+]**
**Gln**	L-Glutamine	$C_5H_{10}N_2O_3$	$[M+H]^+$	*146*, **147**, 148, 149, 150, 151, **152**, *153*	70	17.2	**S5**	**[+]**
**Asp**	L-Aspartate	$C_4H_7NO_4$	$[M+H]^+$	*133*, **134**, 135, 136, 137, **138**, *139*	75	20.2	**S6**	**[+]**
**Pen5P**	Pentose-5-phosphate	$C_5H_{11}O_8P$	$[M-H]^-$	*228*, **229**, 230, 231, 232, 233, **234**, *235*	90	21.4	**S7**	**[-]**
**αKG**	α-Ketoglutarate	$C_5H_6O_5$	$[M-H]^-$	*144*, **145**, 146, 147, 148, 149, **150**, *151*	70	22.1	**S8**	**[-]**
**Mal**	L-Malate	$C_4H_6O_5$	$[M-H]^-$	*132*, **133**, 134, 135, 136, **137**, *138*	80	22.5		**[-]**
**2/3PG**	2/3-Phosphoglycerate	$C_3H_7O_7P$	$[M-H]^-$	*184*, **185**, 186, 187, **188**, *189*	90	23.8	**S9**	**[-]**
**PEP**	2-Phosphoenolpyruvate	$C_3H_5O_6P$	$[M-H]^-$	*166*, **167**, 168, 169, **170**, *171*	70	24.7		**[-]**

Table S4. QTOF-HRMS Parameters (MS Mode and HR Tune) and HILIC Program Settings for Targeted Metabolites and Corresponding ^{13}C-Isotopologues.

Metabolite			***MS and Extraction Parameters (Tune)***			***Program (HILIC)***	***Parameters***
Abbr.	Name	Formula	Ion Species	$^{13}C_n$-Isotopologues (m/z) [u]	ppm	Retention t_R [min]	ESI Polarity
**Phe**	L-Phenylalanine	$C_9H_{11}NO_2$	$[M+H]^+$	**166.0863**, 167.0896, , 168.0930, 169.0963, 170.0997, 171.1030, 172.1064,173.1097,174.1131, 175.1164	10	12.3	**[+]**
**Nva**	L-Norvaline	$C_5H_{11}NO_2$	[M-H]-	**116.0717**	10	14.0	**[-]**
**Pro**	Proline	$C_5H_9NO_2$	$[M+H]^+$	**116.0706**, 117.0740, 118.0773, 119.0807, 120.0840, 121.0874	10	14.2	**[+]**
**Val**	L-Valine	$C_5H_{11}NO_2$	$[M-H]^-$	**116.0717**, 117.0751, 118.0784, 119.0818, 120.0851, 121.0885	10	14.4	**[-]**
**AIBA**	α-Aminobutyrate	$C_4H_9NO_2$	$[M-H]^-$	**102.0561**	10	16.0	**[-]**
**Ala**	L-Alanine	$C_3H_7NO_2$	$[M+H]^+$	**90.0550**, 91.0583, 92.0617, 93.0650	7	17.0	**[+]**
**Gln**	L-Glutamine	$C_5H_{10}N_2O_3$	$[M+H]^+$	**147.0764**, 148.0798, 149.0831, 150.0865, 151.0898, 152.0932	10	18.1	**[+]**
**Ser**	L-Serine	$C_3H_7NO_3$	$[M+H]^+$	**106.0499**, 107.0532, 108.0566, 109.0599	10	18.7	**[+]**
**Glu**	L-Glutamate	$C_5H_9NO_4$	$[M+H]^+$	**148.0604**, 149.0638, 150.0671, 151.0705, 152.0739, 153.0772	100	20.1	**[+]**
**Asp**	L-Aspartate	$C_4H_7NO_4$	$[M+H]^+$	**134.0448**, 135.0481, 136.0515, 137.0548, 138.0582	10	20.5	**[+]**
**Suc**	Succinate	$C_4H_6O_4$	$[M-H]^-$	**117.0190**, 118.0227, 119.0260, 120.0294, 121.0328	20	21.3	**[-]**
**Pen5P**	Pentose-5-phosphate	$C_5H_{11}O_8P$	$[M-H]^-$	**229.0119**, 230.0152, 231.0186, 232.0219, 233.0253, 234.0287	10	21.7	**[-]**
**F6P**	D-Fructose-6-phosphate	$C_6H_{13}O_9P$	$[M-H]^-$	**259.0224**, 260.0258, 261.0292, 262.0325, 263.0359, 264.0392, 265.0426	10	22.1	**[-]**
**αKG**	α-Ketoglutarate	$C_5H_6O_5$	$[M-H]^-$	**145.0142**, 146.0176, 147.0210, 148.0243, 149.0277, 150.0310	10	22.4	**[-]**
**S7P**	D-Sedoheptulose 7-phosphate	$C_7H_{15}O_{10}P$	$[M-H]^-$	**289.033**, 290.0364, 291.0397, 292.0431, 293.0464, 294.0498, 295.0531, 296.0565	10	22.4	**[-]**
**Mal**	L-Malate	$C_4H_6O_5$	$[M-H]^-$	**133.0142**, 134.0176, 135.0210, 136.0243, 137.0277	10	22.7	**[-]**
**G6P**	D-Glucose-6-phosphate	$C_6H_{13}O_9P$	$[M-H]^-$	**259.0224**, 260.0258, 261.0292, 262.0325, 263.0359, 264.0392, 265.0426	10	23.0	**[-]**
**2/3PG**	2/3-phosphoglycerate	$C_3H_7O_7P$	$[M-H]^-$	**184.9857**, 185.9890, 186.9924, 187.9957	10	23.8	**[-]**
**KDPG**	2-dehydro-3-deoxy-6-phospho-D-gluconate	$C_6H_{11}O_9P$	$[M-H]^-$	**257.0068**	10	24.4	**[-]**
**PEP**	2-Phosphoenolpyruvate	$C_3H_5O_6P$	$[M-H]^-$	**166.9751**, 167.9785, 168.9818, 169.9852	10	24.7	**[-]**

Table S5. Regression parameters of HILIC-QQQ-MRM studies of direct and U13C-normalized (IDMS) metabolite-specific signal responsivities using short (up to 1 μM) and extended calibration ranges (up to 400/800 μM, 5 μL sample injection). Supplementary details of Table 1. See Table 3 and S1-4 for the full names of abbreviated metabolites.

**Metabolite**	^{12}C-Calibration (short range up to 1 μM)					^{12}C-Calibration (extended range up to 800 μM)				
Abbr.	Min [nM]	Max [μM]	Slope [Cts/nM]	Intercept 10^3 [Cts]	R^2 [-]	Min [nM]	Max [μM]	Slope [Cts/nM]	Intercept 10^3 [Cts]	R^2 [-]
**Phe**	10	1	1235.7	-1.28	0.9979	10	100	1424.0	725.14	0.9975
**Pro**	50	1	1249.4	35.65	0.9981	50	100	1346.0	1394.50	0.9938
**Val**	200	1	741.2	142.02	0.9981	200	100	702.9	1008.17	0.9955
**Ala**	50	1	262.2	27.29	0.9982	50	400	243.9	1363.90	0.9905
**Gln**	50	1	139.9	4.10	0.9992	50	800	144.1	1418.62	0.9919
**Ser**	100	1	119.9	30.76	0.9987	100	600	120.2	852.27	0.9932
**Glu**	50	1	159.1	33.36	0.9995	50	400	188.6	362.43	0.9983
**Asp**	25	1	76.8	4.86	0.9995	25	400	100.7	67.19	0.9994
**Suc**	100	1	21.1	0.32	0.9884	100	800	18.1	206.91	0.9914
**Pen5P***	25	1	81.2	3.47	0.9997	25	20	66.1	23.14	0.9949
**F6P**	50	1	43.8	0.47	0.9981	50	400	37.5	44.43	0.9907
**αKG**	50	1	127.5	3.46	0.9997	50	10	34.6	248.22	0.9903
**S7P**	50	1	62.5	-0.75	0.9966	50	600	34.7	360.28	0.9914
**Mal**	50	1	102.9	1.99	0.9977	50	40	93.2	48.51	0.9946
**G6P**	50	1	30.0	0.80	0.9996	50	800	29.8	146.41	0.9988
**PEP**	50	1	155.9	0.98	0.9995	50	4	138.7	9.20	0.9975

**Metabolite**	U^{13}C-IDMS (short range up to 1 μM)					U^{13}C-IDMS (extended range up to 800 μM)				
Abbr.	Min [nM]	Max [μM]	Slope 10^{-3} [-/nM]	Intercept 10^{-3} [-]	R^2 [-]	Min [nM]	Max [μM]	Slope 10^{-3} [-/nM]	Intercept 10^{-3} [-]	R^2 [-]
**Phe**	10	1	0.795	-1.537	0.9972	10	800	0.820	5662.9	0.9954
**Pro**	10	1	0.094	2.560	0.9975	10	800	0.087	707.3	0.9948
**Val**	200	1	0.238	43.077	0.9973	200	800	0.245	1003.0	0.9986
**Ala**	50	1	0.011	1.231	0.9979	50	800	0.015	22.7	0.9991
**Gln**	50	1	0.007	0.216	0.9991	50	800	0.010	-3.6	0.9997
**Ser**	100	1	0.210	55.294	0.9988	100	800	0.298	-341.4	0.9999
**Glu**	50	1	0.001	0.317	0.9995	50	400	0.002	-0.7	0.9999
**Asp**	25	1	0.055	3.687	0.9995	25	400	0.084	-123.2	0.9998
**Suc**	100	1	0.003	0.045	0.9880	100	800	0.004	-15.6	0.9995
**Pen5P***	25	1	0.207	8.418	0.9997	25	400	0.284	-103.3	0.9998
**F6P**	50	1	0.058	0.321	0.9964	50	800	0.098	-360.3	0.994
**αKG**	50	1	0.238	2.934	0.9989	50	800	0.388	-1298.8	0.9996
**S7P**	50	1	0.392	-4.216	0.9966	50	800	0.650	-2074.2	0.9996
**Mal**	50	1	0.021	0.376	0.9973	50	800	0.036	-174.6	0.999
**G6P**	50	1	0.030	0.808	0.9995	50	800	0.050	-164.9	0.9997
**PEP**	50	1	0.324	-1.745	0.9980	50	800	0.492	-1216.0	0.9998

Pen5P = Pooled Ribose- and Ribulose-5-Phosphate

Table S6. Regression parameters of HILIC-QTOF-HRMS studies of direct and U13C-normalized (IDMS) metabolite-specific signal responsivities using short (up to 1 µM) and extended calibration ranges (up to 400/800 µM, 5 µL sample injection). Supplementary details of Table 1. See Table 3 and S1-4 for the full names of abbreviated metabolites.

**Metabolite**	**^{12}C-Calibration** (short range up to 1 µM)					**^{12}C-Calibration** (extended range up to 800 µM)				
Abbr.	Min [nM]	Max [µM]	Slope [Cts/nM]	Intercept 10^3 [Cts]	R^2 [-]	Min [nM]	Max [µM]	Slope [Cts/nM]	Intercept [10^3 Cts]	R^2 [-]
**Phe**	50	1	357.8	0.16	0.9990	50	40	328.9	67.53	0.9995
**Pro**	50	1	237.9	-0.03	0.9910	50	100	141.5	263.79	0.9943
**Val**	400	1	131.2	44.62	0.9967	400	40	95.6	149.68	0.9949
**Ala**	50	1	27.7	2.66	0.9972	50	800	27.9	100.89	0.9967
**Gln**	50	1	41.0	-0.47	0.9978	50	400	28.0	175.31	0.9900
**Ser**	100	1	22.7	1.48	0.9955	100	800	22.8	19.22	0.9999
**Glu**	50	1	100.3	24.09	0.9986	50	200	72.6	223.20	0.9922
**Asp**	200	1	51.3	-6.68	0.9949	200	200	62.3	16.24	0.9991
**Suc**	200	1	172.5	-2.47	0.9971	200	800	77.5	1116.90	0.9950
**Pen5P***	200	1	218.8	3.87	0.9911	200	15	201.8	57.12	0.9938
**F6P**	200	1	134.1	-5.69	0.9995	200	40	84.0	101.18	0.9923
**αKG**	50	1	954.3	38.51	0.9991	50	2	876.3	65.30	0.9969
**S7P**	100	1	104.4	-1.19	0.9963	100	30	85.6	41.94	0.9943
**Mal**	50	1	678.4	30.27	0.9981	50	30	508.5	315.81	0.9940
**G6P**	200	1	14.7	1.60	0.9228	200	800	22.7	-109.92	0.9964
**PEP**	100	1	641.2	7.43	0.9997	100	10	448.3	161.14	0.9905
**Metabolite**	**U^{13}C-IDMS** (short range up to 1 µM)					**U^{13}C-IDMS** (extended range up to 800 µM)				
Abbr.	Min [nM]	Max [µM]	Slope 10^{-3} [-/nM]	Intercept 10^{-3} [-]	R^2 [-]	Min [nM]	Max [µM]	Slope 10^{-3} [-/nM]	Intercept 10^{-3} [-]	R^2 [-]
**Phe**	50	1	0.743	-2.068	0.9998	50	800	0.754	528.8	0.9996
**Pro**	50	1	0.101	0.195	0.9946	50	800	0.104	-72.1	0.9997
**Val**	400	1	0.231	86.447	0.9889	400	800	0.275	-1895.5	0.9942
**Ala**	50	1	0.015	1.336	0.9971	50	800	0.017	-39.9	0.9986
**Gln**	50	1	0.010	-0.146	0.9985	50	800	0.012	-40.9	0.9989
**Ser**	100	1	0.244	13.764	0.9946	100	800	0.263	-502.4	0.9993
**Glu**	50	1	0.002	0.466	0.9978	50	400	0.002	-3.6	0.9991
**Asp**	200	1	0.061	-7.772	0.9956	200	400	0.077	-154.3	0.9991
**Suc**	200	1	0.005	-0.069	0.9963	200	800	0.004	-7.7	0.9990
**Pen5P***	200	1	0.219	2.233	0.9927	200	400	0.206	262.1	0.9983
**F6P**	200	1	0.074	-3.840	0.9970	200	800	0.067	-89.1	0.9990
**αKG**	50	1	0.265	6.055	0.9976	50	800	0.300	-1258.0	0.9985
**S7P**	100	1	0.430	-6.924	0.9963	100	800	0.413	1033.1	0.9954
**Mal**	50	1	0.028	1.079	0.9978	50	800	0.036	-104.9	0.9996
**G6P**	200	1	0.037	-0.913	0.9926	200	800	0.040	-97.7	0.9944
**PEP**	100	1	0.370	-1.646	0.9975	100	800	0.385	-576.9	0.9990

Pen5P = Pooled Ribose- and Ribulose-5-Phosphate
